# Plasma D-Dimer Level Correlates with Age, Metastasis, Recurrence, Tumor-Node-Metastasis Classification (TNM), and Treatment of Non-Small-Cell Lung Cancer (NSCLC) Patients

**DOI:** 10.1155/2021/9623571

**Published:** 2021-10-19

**Authors:** Jiqiang Guo, Ying Gao, Zhihua Gong, Pengfei Dong, Yajie Mao, Fang Li, Jianrong Rong, Junping Zhang, Yongnian Zhou, Huijing Feng, Hongxia Guo, Linxia Gu, Meiwen An, Kaixue Wen, Jin Zhang

**Affiliations:** ^1^College of Biomedical Engineering, Taiyuan University of Technology, Taiyuan, 030024, Shanxi, China; ^2^Shanxi Bethune Hospital, Shanxi Academy of Medical Sciences, Tongji Shanxi Hospital, Third Hospital of Shanxi Medical University, Taiyuan, 030032, China; ^3^Tongji Hospital, Tongji Medical College, Huazhong University of Science and Technology, Wuhan, 430030, China; ^4^Department of Biomedical and Chemical Engineering and Sciences, Florida Institute of Technology, Melbourne, 32901 FL, USA

## Abstract

**Objective:**

This study is aimed at teasing out the correlation of plasma D-dimer (D-D) levels to age, metastasis, TNM stage (tumor-node-metastasis classification), and treatment in non-small-cell lung cancer (NSCLC) patients of different ages, to facilitate early diagnosis of hypercoagulable state, choose appropriate treatment, and use appropriate anticoagulants. Hence, thrombosis and complications caused by excessive anticoagulants can be prevented; thrombus or disseminated intravascular coagulation (DIC) and other complications in elderly patients with NSCLC can be reduced or avoided. By monitoring the level of plasma D-D in patients with NSCLC, recurrence and metastasis can be predicted in the early stage and the TNM stage can be evaluated.

**Methods:**

A total of 670 patients with NSCLC were selected in Shanxi Bethune Hospital from March 2014 to October 2020 as the experimental group, and 950 healthy people were selected from the physical examination center of the same hospital as the control group. The data of patients with NSCLC diagnosed for the first time without any treatment were collected and grouped based on metastasis, TNM stage, treatment, and pathological type, and the correlation with plasma D-D level was analyzed. Plasma D-D levels were measured by immunoturbidimetry on an ACL TOP 700 Automatic Coagulation Analyzer. The patients were further divided into two groups according to different treatment methods, and the differences in plasma D-D levels between patients receiving chemotherapy and those receiving targeted therapy in different treatment cycles were analyzed. The correlation between D-D levels and age in healthy controls was analyzed. The difference in D-D levels between NSCLC patients and healthy controls of the same age was analyzed.

**Results:**

All data of both the experimental group and the control group were normally distributed. The average age of the experimental group was 61.31 ± 6.23 (range: 36-92) years. The average age of the control group was 61.14 ± 11.12 (range: 35-85) years. There was no significant difference in gender between the experimental group and the control group (*p* > 0.05). The plasma D-D level of NSCLC patients was significantly higher than that of the healthy controls (*p* < 0.05). No significant difference in plasma D-D level was found between NSCLC patients of different genders, and the finding was similar between healthy controls of different genders (*p* > 0.05). Significant difference in the D-D level was found between the groups of 30-59 years and 60-69 years (*p* < 0.05), between groups of 60-69 years and 70-79 years (*p* < 0.05), and between 70-79 years and ≥80 years (*p* < 0.05). The plasma D-D level of patients ≤ 79 years old increased with age, but it decreased in those over 80 years old. According to Pearson correlation analysis, there was a positive correlation between the D-D level and the age of NSCLC patients under 79 years old (*p* < 0.05). The differences in D-D levels between the four age groups were statistically significant (*p* < 0.05), showing an upward trend of the D-D level in healthy controls with the increase of age. There were statistically significant differences in D-D levels between NSCLC patients and healthy controls of the matching age group (*p* < 0.05), suggesting that NSCLC patients had significantly higher D-D levels than healthy people of the same age group. The differences in D-D levels between NSCLC patients without metastasis, NSCLC patients with metastasis, and healthy people were statistically significant (*p* < 0.05). The patients with metastasis had the highest D-D level, and healthy people had the lowest D-D level. The difference in plasma D-D levels between patients of different TNM stages was statistically significant (*p* < 0.05). Patients with an advanced TNM stage tended to have higher D-D levels. The TNM stage and D-D level of NSCLC patients changed significantly before and after treatment. An earlier stage was related to a more obvious change in D-D levels after treatment with a statistically significant difference (*p* < 0.05). A more advanced stage was associated with a smaller change in the D-D level after treatment, with no statistically significant difference (*p* > 0.05). The plasma D-D levels before and after four cycles of chemotherapy or targeted therapy were higher than those of the healthy control group, and the differences were statistically significant (*p* < 0.05). The D-D level of patients after chemotherapy was significantly lower than that before chemotherapy (*p* < 0.05), but there was no significant difference before and after targeted therapy (*p* > 0.05). The D-D level after the first cycle of chemotherapy was higher than that before chemotherapy. The level of D-D after the third and fourth cycles was significantly lower than that before chemotherapy (*p* < 0.05). No significant difference was found between the D-D level before treatment and that after four cycles of chemotherapy (*p* > 0.05).

**Conclusion:**

It is suggested that coagulation test indexes should be included to evaluate the treatment regimen for NSCLC patients. Most patients with NSCLC are in a hypercoagulable state, which is related to age, tumor invasion and metastasis, recurrence, and treatment. Regular monitoring of plasma D-D levels can facilitate early diagnosis of a hypercoagulable state and timely and appropriate use of anticoagulants, to avoid or reduce complications such as venous thromboembolism in NSCLC patients and to prevent the risk of bleeding caused by excessive anticoagulants. Clinicians can choose the treatment with less harm and maximum benefit for NSCLC patients based on the plasma D-D level. When in a hypercoagulable state, the body's blood viscosity increases, making it more conducive to the growth and infiltration of tumor cells. Our study shows that the recurrence and metastasis of NSCLC are related to coagulation indexes, which provides a theoretical basis for the early diagnosis and treatment of recurrent and metastatic NSCLC.

## 1. Introduction

Lung cancer is the most common and deadly malignant tumor worldwide. At the time of diagnosis, 80% of lung cancer patients are in the advanced stage, and the 5-year survival rate is less than 15%, posing a serious threat to human health [1, 2]. Non-small-cell lung cancer (NSCLC), the most common pathological type [3] of lung cancer, includes squamous cell carcinoma, adenocarcinoma, adenosquamous cell carcinoma, and other histological types except small-cell carcinoma and accounts for about 85% of all lung cancer cases [4]. Compared with small-cell carcinoma, NSCLC features slow growth and division, as well as late diffusion and metastasis.

The hypercoagulable state, which is common in patients with malignant tumors, can result in thrombosis, such as pulmonary embolism, and hence is a leading factor of death [5–7]. In recent years, studies have found that the disordered coagulation function may be related to the occurrence and development as well as the clinical treatment and prognosis of malignant tumors [8, 9]. Therefore, the changes in coagulation function and the necessity of early anticoagulation therapy have attracted the attention of clinicians globally [10, 11]. The complex mechanism of hypercoagulable state in patients with malignant tumors is closely related to the biological characteristics of the tumor and can lead to disordered coagulation and fibrinolysis system [12]. Tumor cells can secrete more tissue factors, promote the release of procoagulant substances, and interact with vascular endothelial cells, platelets, and monocytes, which all contribute to the hypercoagulable state. Studies have shown that fibrinogen (FIB), platelet (PLT), and cancer cells can form microthrombosis under a hypercoagulable state, so tumor cells can escape immune attack, thus leading to tumor metastasis [13, 14]. In this paper, by analyzing the characteristics of D-D levels in NSCLC patients, we retrospectively explored the correlation of D-D levels to age, metastasis, TNM stage, and treatment methods, as well as the effect of D-D levels on a hypercoagulable state in NSCLC patients.

## 2. Materials and Methods

### 2.1. Population Statistics

1. 670 NSCLC cases and 950 healthy volunteers were selected for the investigation. All of them were in the Shanxi Bethune Hospital from March 2014 to October 2020. The NSCLC patients were diagnosed according to the World Health Organization criteria (2015 edition). And their TNM staging is based on the International Association for the Study of Lung Cancer (IASLC) and the Union for International Cancer Control (UICC) (issued in January 2017) (Table 1). Healthy volunteers mean they received physical examination without obvious heart, liver, or kidney diseases and the normal result of accessory examination
2. NSCLC patients of inclusive and exclusive criteria: inclusive: age group: 35 to 95 years old, male or female; firstly diagnosed by pathology or cytology; expected survival life-time ≥ 3 months; without other cancers; volunteer; and no enrollment in other clinical trials. Exclusive: with severe immunodeficiency or autoimmune diseases; with chronic congestive heart failure; with a history of operation, trauma, and burn in six months; and with hormone or anticoagulant therapies
3. TNM staging

(4) NSCLC patient therapy grouping

Chemotherapy group: chemotherapy regimen: on the basis of supporting treatment, pemetrexed (MTA, 500 mg/bottle, Hausen Pharmaceutical, H20093996) 500 mg/m^2^ (d1) + cisplatin (DDP, 20 mg/bottle, Qilu Pharmaceutical, production batch number: 09100212) 75 mg/m^2^ (d1-d5), ivgtt, was given according to the body surface area. Oral folic acid 400 *μ*g/d was given 7 days before the first administration of pemetrexed for at least 5 days until 21 days after the last administration, and vitamin B12 1000 *μ*g was injected intramuscularly 7 days before the beginning of the first cycle of chemotherapy, then once every 3 cycles. Dexamethasone tablets (4 mg, bid) were taken orally on the day before, the same day, and after administration to prevent skin reaction. The above chemotherapy consists of one cycle every 3 weeks, with a total of four cycles. If the disease progressed or there were serious adverse reactions, the treatment was stopped and the study was withdrawn, but the adverse reactions were recorded. Targeted drug therapy group: the targeted treatment group was treated with erlotinib (Roche Registration Ltd., H20170030 (150 mg/d)). After continuous treatment for 3 weeks, the interval was 1 week for 1 cycle, and the treatment was carried out for 4 cycles.

(5) Healthy volunteers of inclusive criteria

Age group: 35 to 95 years old, male or female; Negative tumor markers; Healthy (conclusion) with the normal result of accessory examination; Volunteer; No enrollment in other clinical trials.

(6) Age grouping (NSCLC cases and 950 healthy volunteers)

### 2.2. Methods

Plasma sample: fasting elbow venous blood was collected (containing sodium citrate anticoagulant; anticoagulant and whole blood ratio was 1 : 9) and centrifugated at 3500 r/min for 10 min (*r* = 13 cm), 25°C. NSCLC patient's blood samples were collected 12 hours before and after treatment.

Plasma PT, INR, APTT, TT, FIB-C, AT-III, and D-D were detected by an ACL TOP 700 Automatic Coagulation Analyzer. The test kit was purchased from Wofen company of Spain (modelRecombiplasTin 2G, RecombiplasTin 2G, APTT-SP, Thrombin Time, Fibrinogen-C (clauss method) XL Kit, Liquid Antithrombin, D-D HS500). The normal range of Pt was 9 s-12 s; the normal range of PTA was 80%-160%; the normal range of INR was 0.8-1.4; the normal range of APTT was 28 s-41 s; the normal range of FIB-C was 2.00 g/l-4.40 g/l; the normal range of D-D was 0-200 ng/l.

### 2.3. Internal Quality Control Results of Coagulation Test Items

#### 2.3.1. Statistical Analysis

All data were analyzed by SPSS 19.0 statistical software, and the measurement data in accordance with normal distribution were analyzed by means ± standard deviation$\left(\bar{x}\pm s\right)$ description and comparison between groups using the *t*/*F* test. The qualitative data (or counting data) were described by percentage, and the comparison between groups was described by percentage *χ*^2^ inspection. Pearson correlation was used to analyze the correlation. The difference was statistically significant (*p* < 0.05).

## 3. Results

### 3.1. Difference in the D-D Level between the Experimental Group and the Control Group

There were 358 male and 312 female patients in the NSCLC group. The age ranged from 36 to 92 years, with an average of 61.31 ± 6.23 years. There were 497 males and 453 females in the control group, with an average age of 60.21 ± 11.12 years. The experimental group and the control group were further divided into four age groups (30-59, 60-69, 70-79, and ≥80 years), respectively. There was no significant difference in the number of different genders between the two groups (*p* > 0.05). A significant difference was found in the number of cases of different ages between the two groups (*p* < 0.05), which was comparable. Compared with the control group, the levels of D-D (D-dimer), PT (Prothrombin Time), INR (International Normalized Ratio), and FIB-C (Fibrinogen-c) in the experimental group were significantly higher (*p* < 0.05), the level of TT (Thrombin Time) was significantly lower (*p* < 0.05), and the levels of PT% (Prothrombin Time Activity) and APTT (Activated Partial Thromboplastin Time) were significantly lower (*p* > 0.05) (Tables 2-5).

### 3.2. Difference in D-D Levels of Different Age Groups in the Experimental Group and the Control Group

The data of the experimental group were normally distributed. The difference in D-D level between the 30-59 age group and the 70-79 age group was statistically significant (*p* < 0.05), and no significant difference was found between the other age groups (*p* < 0.05). The D-D levels of 30-59, 60-69, and 70-79 age groups showed an overall upward trend with increasing age, but the D-D level of the ≥80 age group was lower than that of the 60-69 age group and the 70-79 age group. Pearson correlation analysis showed that the D-D levels of 30-59, 60-69, and 70-79 age groups were positively correlated to age (*p* < 0.05), indicating that in patients younger than 80 years, D-D levels presented an upward trend with the increase of age (Table 6).

The data of the control group followed a normal distribution. The between-group difference in the D-D level was statistically significant in all age groups (*p* < 0.05). Pearson correlation analysis showed a positive correlation between D-D level and age in all age groups (*p* < 0.05). The level of D-D in the control group showed an upward trend with the increase of age in the controls younger than 80 years old (Table 6).

The D-D level of the experimental group was significantly higher than that of the control group (*p* < 0.05). The D-D level in all age groups of the experimental group was significantly higher than that in all age groups of the control group. The D-D level showed an upward trend with the increase of age in subjects younger than 80 years old in both groups. However, for those older than 80 years in the experimental group, the D-D level decreased (Table 6).

### 3.3. Difference in D-D Levels between Patients with and without Metastasis

There were 98 patients without metastasis and 572 patients with metastasis. The D-D level of patients without metastasis was significantly higher than the control group (*p* < 0.01). Therefore, it was the D-D level in patients with metastasis (*p* < 0.01). The D-D level of patients without metastasis was significantly higher than that of those with metastasis (*p* < 0.01) (Table 7).

**Figure 1:**
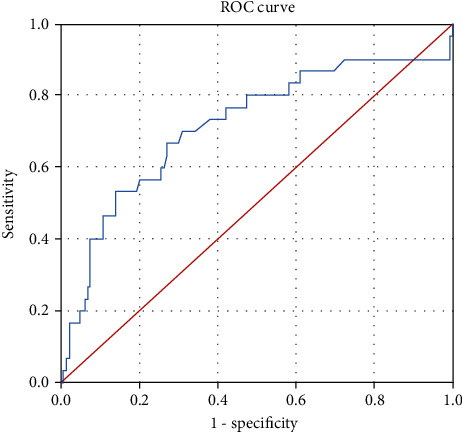
ROC curves of D-dimer for predicting tumor metastasis in NSCLC patients.

ROC curves of the D-D level for predicting tumor metastasis in NSCLC patients showed an area under the curve of 0.717 (95% confidence interval, 0.603-0.831, *p* < 0.001) (Figure 1).

### 3.4. Difference in D-D Levels between Patients in Different TNM Stages

According to the TNM classification: 98 cases were in stage I, 101 cases in stage II, 172 cases in stage III, and 299 cases in stage IV. Compared with the D-D level of the control group, the D-D levels of the patients in all TNM stages were higher than those of the control group. And a more advanced TNM stage was related to a higher D-D level. There was no statistical significance between stage I patients and the control group (*p* > 0.05), but a statistically significant difference was found when comparing the D-D level of the control group with that of stages II, III, and IV patients (*p* < 0.05). There was a significant difference in the D-D level between stage I and stage II patients (*p* < 0.05) (Table 8). The D-D level of stage III patients was significantly higher than that of stage I and II patients (*p* < 0.05) (Table 8). The D-D level of stage IV patients was significantly higher than that of stage I, II, and III patients (*p* < 0.05) (Table 9). Pearson correlation analysis showed the D-D levels of patients were positively correlated with stage (*p* < 0.05), indicating that the D-D level presented an upward trend with the increase of TNM classification (Table 9).

### 3.5. Differences in D-D Levels of the Experimental Group with Different Treatment Methods before and after Treatment and Comparison with the Control Group

The D-D level of the experimental group after treatment (chemotherapy or targeted therapy) was significantly lower than that before treatment (*p* < 0.05) (Table 10). However, despite the type of treatment used, the D-D level after treatment was still higher than that in the control group, with a significant difference (*p* < 0.05) (Table 11).

For patients receiving chemotherapy, the D-D level before treatment was significantly higher than that of the control group (*p* < 0.05); the D-D level after treatment was significantly lower than that before treatment (*p* < 0.05) (Table 12). The D-D level at each chemotherapy cycle was lower than that before treatment, and the D-D level at the third cycle was significantly different from that before the treatment (*p* < 0.05) (Table 13). Pearson correlation analysis showed that the D-D levels of patients were negatively correlated to the cycle (*p* < 0.05), indicating that D-D levels presented a downward trend with the increase of chemotherapy cycle (Table 13). The difference in the D-D level between cycles was statistically significant (*p* < 0.05) (Table 14).

For patients receiving targeted therapy, the D-D level before treatment was significantly higher than that of the control group (*p* < 0.05) (Table 15). The D-D level decreased after treatment compared with that before the treatment, but the difference was not significant (*p* > 0.05) (Table 15). The D-D level at each treatment cycle was lower than that before the treatment. Although not significant, the difference was more obvious in the later cycle of treatment (*p* > 0.05) (Table 16). The comparison of D-D levels between the treatment cycles showed that the decrease of the D-D level was more obvious in the later cycle of treatment (*p* > 0.05) (Table 17). Pearson correlation analysis showed that the D-D levels of patients were negatively correlated to the cycle (*p* < 0.05), indicating that D-D levels presented a downward trend with the increase of targeted therapy cycles (Table 16).

## 4. Discussion

The incidence of NSCLC ranks first among all tumors, and it is more commonly found in the elderly [15]. However, in recent years, patients suffering NSCLC tend to be younger [16]. Due to the lack of typical clinical symptoms, NSCLC cases may have missed or delayed diagnosis. When diagnosed, they are already in the late stage. D-D, a degradation product of cross-linked fibrin, is a molecular marker that can reflect the hypercoagulable state and hyperfibrinolysis in the body. The increase of D-D often indicates active thrombosis and fibrinolysis in the body. D-D has a high negative predictive value in the exclusion of thrombotic diseases. When D-D is lower than the clinical decision level, the possibility of thrombosis can be excluded.

Studies have shown that the survival rate of malignant tumor patients without venous thromboembolism (VTE) is at least 3 times higher than that of patients with VTE, and the risk of death for tumor patients with VTE can be increased by 2-6 times [17]. The incidence of VTE in NSCLC patients is about 4%-10%. In patients with advanced NSCLC, about 10% have symptomatic VTE, and more than 50% have asymptomatic VTE [18]. VTE, a common complication and the second cause of death in NSCLC patients, is associated with a poor prognosis of NSCLC. The hypercoagulable state of blood is the underlying mechanism for VTE. Therefore, the change of coagulation function in patients with NSCLC is of great value in predicting survival and guiding treatment.

Age is often used as an important reference index. Studies on the relationship between D-D levels and age in healthy people have shown that healthy elderly people (≥60) have a significantly higher D-D level than healthy young people [19]. The increase of D-D levels in the elderly may be caused by the elevated probability of vascular sclerosis, vulnerable vascular endothelial cells, decreased inhibition of coagulation system activation, secondary hyperfibrinolysis, and increased risk of hypertension and hyperlipidemia. D-D is mainly excreted by the kidney and cleared by phagocytes. As the body's metabolic capacity declines with the increase of age, the D-D level will elevate [20]. In line with the above conclusions, the results of this study reveal the correlation between age and D-D level. With the increase of age, the D-D level shows an upward trend, which is also consistent with the conclusion of Bacon et al.'s study [21]. However, the mechanism of this increase is not clear. Studies have shown that in NSCLC patients, older age is associated with a later TNM stage, lower physical condition score, higher plasma D-D level, and shorter survival time [22]. This study demonstrates that the D-D level of elderly NSCLC patients is significantly higher than that of healthy people of all age groups, indicating that the coagulation and fibrinolysis system is activated in elderly NSCLC patients. In NSCLC patients of 50-79 years old, as age increases, the D-D level is positively correlated with age. Elderly patients have increased risk of hypertension, cardiovascular diseases, and diabetes, and the development of these diseases may damage the endothelial cells, which is conducive to the formation of intravascular thrombosis [23, 24]. And this formation is manifested by the rise of the D-D level. However, considering the weakened function of the major organs in NSCLC patients over 80 years, together with complications and other influencing factors, NSCLC patients over 80 years old are usually excluded from the study or the number of such patients enrolled is very small [25]. Hence, this study only applied Pearson correlation analysis to patients of 50-79 years old for correlation analysis.

Increasingly clinical evidence has shown obvious abnormalities in the coagulation system and significantly increased D-D levels in patients with malignant tumors, such as digestive system malignant tumor, liver cancer, colorectal cancer, gastric cancer, and breast cancer. The mechanism may be that tumor cells or necrotic surrounding tissues trigger the release of tissue factors, which activate coagulation factors X and XI in the extrinsic coagulation system and fibrinolytic system, leading to the local production of fibrinolytic enzymes, which can directly degrade the extracellular matrix and endow the tumor cells with the invading and metastatic abilities [26]. In this process, the released inflammatory mediators (such as IL and TNF-*α*) damage the vascular endothelial cells, resulting in the hypercoagulable state of blood [27, 28] and making tumor cells adhere to the vascular wall. The hypercoagulable state in tumor patients not only increases the risk of VTE but also promotes blood vessel distribution around the tumor, enriches the blood supply of tumor tissues, and enhances tumor progression (recurrence and metastasis) [29]. As a specific degradation product of fibrin monomers cross-linked by activator XIII and then hydrolyzed by fibrinolytic enzymes, D-D is a specific marker of fibrinolysis. Continuous increase of D-D often indicates disease progression or poor prognosis.

This study found that the D-D level in NSCLC patients was significantly higher than that in the control group, which can be explained as follows. The tissue of NSCLC patients can secrete procoagulants, and their antithrombotic ability decreases after the vascular intima is infiltrated by lung cancer tissue, both resulting in hypercoagulability in patients [30]. Cancer cells in the blood of NSCLC patients can directly elevate the D-D level, and reactive white blood cells can also increase the plasma D-D level. The TNM stage is an important index for the prognosis and treatment of tumors [31]. Our study found that the TNM stage was significantly correlated with the plasma D-D level. An advanced TNM stage was associated with increased D-D levels. The D-D level in stage III and IV patients of NSCLC was significantly higher than that in patients of stages I and II, with statistical significance (*p* < 0.05). There was no significant difference in the level of D-D between stage I and stage II patients (*p* > 0.05). The D-D level of stage III patients was significantly higher than that of stage I and II patients (*p* > 0.05). The main reason may be that the tumor infiltration of stage III and IV patients is deeper and more extensive than that of stage I and II patients and is often accompanied by lymph nodes and other distant metastases. Cancer patients often suffer complications, such as hypertension, hyperlipidemia, heart failure, and myocardial infarction, which can accelerate blood coagulation, making the D-D level higher. Therefore, monitoring D-D levels and improving the hypercoagulable state of blood in NSCLC patients of different TNM stages can facilitate the early prevention and treatment so as to increase the survival rate of patients. In addition, a majority of NSCLC patients are elderly, and elderly patients, especially those with advanced cancer, are usually bedridden for a long time, resulting in blood stasis. Therefore, NSCLC patients are more likely to suffer abnormal coagulation function and thrombosis than healthy people [32].

NSCLC is a malignant tumor with high mortality. In clinical practice, thrombolysis, anticoagulation, and other treatments usually start after obvious thrombosis appears in patients, and therefore, the curative effect is poor. Many patients die of serious thromboembolism, disseminated intravascular coagulation, and other adverse events [33]. Chemotherapy and targeted therapy, which can improve the clinical symptoms of patients, are commonly used to treat NSCLC [34]. Since VTE directly impacts the survival of NSCLC patients, and the best monitoring index of VTE is the D-D level, the effect of chemotherapy and targeted therapy on D-D levels in NSCLC patients is worth further studying.

This study found that the plasma D-D level of NSCLC patients was higher than that of healthy people, with significant differences, indicating that NSCLC patients are in a hypercoagulable state, which can cause VTE at any time [35]. This result also suggests the necessity to monitor and control the plasma D-D level in the follow-up treatment of NSCLC patients to prevent thrombosis.

This study also found that after chemotherapy, the difference in the D-D level between two consecutive cycles was not significant, but a significant difference was found between the two cycles with a one-cycle interval, and the difference before and after treatment was also significant, indicating that chemotherapy can well control the hypercoagulable state of NSCLC patients. In patients receiving targeted therapy, the D-D level showed an overall downward trend after treatment. Although there was no significant difference in the first four cycles, the difference was increasingly obvious. Our results suggest that targeted therapy has an impact on the D-D level of NSCLC patients, but chemotherapy exerts a better effect on the improvement of hypercoagulable state in patients. In addition, heparin can be administered when necessary based on the D-D level of patients [36] in order to achieve an ideal therapeutic effect.

## 5. Conclusion

The plasma D-D level is conducive to the early detection and treatment of NSCLC. For NSCLC patients, blood coagulation function should be examined regularly to detect the hypercoagulable state of blood and conduct treatment timely. An older age is associated with a higher D-D level, advanced stage, and metastasis in NSCLC patients. The elevated D-D level indicates the existence of a hypercoagulable state and the activation of the secondary fibrinolytic system, which can increase the risk of VTE and disseminated intravascular coagulation. Therefore, monitoring the D-D level can help to judge and predict the disease condition. To treat NSCLC, we can use antithrombotic, low molecular weight heparin, and other preventive anticoagulants to eliminate or reduce the risk of deep venous thrombosis and other complications and to control tumor metastasis and improve the comprehensive curative effect and survival of patients.

## Figures and Tables

**Table 1:** TNM classification of non-small-cell lung cancer (8th edition).

Comprehensive staging	T stage	N stage	M stage	Number
0 stage	Tis (carcinoma in situ)	N0	M0	0
IA1 stage	T1a (mis)	N0	M0	98
IA2 stage	T1b	N0	M0	
IA3 stage	T1c	N0	M0	
IB stage	T2a	N0	M0	
IIA stage	T2b	N0	M0	101
IIB stage	T1a-c	N1	M0	
	T2a	N1	M0	
	T2b	N1	M0	
	T3	N0	M0	
IIIA stage	T1a-c	N2	M0	172
	T2a-b	N2	M0	
	T3	N1	M0	
	T4	N0	M0	
	T4	N1	M0	
IIIB stage	T1a-c	N3	M0	
	T2a-b	N3	M0	
	T3	N2	M0	
	T4	N2	M0	
IIIC stage	T3	N3	M0	
	T4	N3	M0	
IVA stage	Any T	Any N	M1a/b	299
IVB stage	Any T	Any N	M1c	
Total				670

TNM:Tumor Node Metastasis.

**Table:** (a) Comparison of the number of patients in different gender groups between the experimental group and the control group (*n*)

Grouping	Experience group	Control group	*χ* ^2^ value	*p* value
Male	358	497	0.197	0.657
Female	312	453		
Total	670	950		

**Table:** (b) Comparison of the number of patients in different age groups between the experimental group and the control group (*n*)

Grouping (year)	Experience group	Control group	*χ* ^2^ value	*p* value
30-59	189	349	26.815	*p* ≤ 0.001
60-69	273	283		
70-79	167	177		
≥80	41	141		
Total	670	950		

**Table 3:** Internal quality control results of coagulation test items from 2014 to 2020.

Year	PT		INR		APTT		TT		FIB-C		AT-III		D-D	
	IQC	CV (%)	IQC	CV (%)	IQC	CV (%)	IQC	CV (%)	IQC	CV (%)	IQC	CV (%)	IQC	CV (%)
2020	11.05 + 0.46	4.29	1.01 + 0.05	4.14	30.29 + 0.95	3.81	14.41 + 0.78	4.62	2.85 + 0.13	4.56	102.00 + 3.89	3.44	421.00 + 20.00	4.75
2019	11.50 + 0.50	4.35	1.08 + 0.04	3.70	30.70 + 1.25	4.07	14.50 + 0.79	4.83	3.04 + 0.15	4.93	110.00 + 4.42	4.17	421.00 + 20.00	4.75
2018	11.00 + 0.50	4.50	1.02 + 0.05	4.90	30.30 + 1.20	3.96	14.80 + 0.72	4.73	3.19 + 0.13	4.08	105.00 + 4.11	3.91	386.00 + 22.00	5.70
2017	11.50 + 0.45	3.91	1.06 + 0.05	4.72	31.00 + 1.12	3.61	14.60 + 0.83	5.68	3.30 + 0.16	4.85	103.00 + 3.98	3.81	354.00 + 22.00	5.25
2016	11.50 + 0.50	4.35	1.07 + 0.06	5.59	31.08 + 1.30	4.18	14.35 + 0.66	5.53	3.29 + 0.15	4.56	108.00 + 4.22	4.16	753.00 + 23.00	3.12
2015	10.90 + 0.50	4.59	0.99 + 0.04	5.06	31.40 + 0.99	3.87	14.71 + 0.59	4.23	3.04 + 0.19	5.25	110.00 + 4.31	4.07	728.00 + 24.00	3.23
2014	11.50 + 0.66	4.74	1.06 + 0.06	5.66	30.90 + 1.56	5.05	14.63 + 0.56	3.89	2.85 + 0.19	6.67	103.00 + 3.76	4.05	739.00 + 25.00	3.34

IQC: internal quality control.

**Table 4:** Comparison of D-D between patients with non-small-cell lung cancer and normal people $\left(\bar{x}\pm \text{s}\right)$.

Test items	Experience group	Control group	*t* value	*p* value
PT	11.36 ± 1.01	10.87 ± 0.65	4.452	*p* ≤ 0.001
INR	1.05 ± 0.09	1.01 ± 0.06	4.503	*p* ≤ 0.001
APTT	31.65 ± 2.71	31.46 ± 3.47	0.481	0.631
TT	13.58 ± 1.11	14.22 ± 1.22	-4.269	*p* ≤ 0.001
FIB-C	4.00 ± 1.03	3.00 ± 0.55	9.282	*p* ≤ 0.001
AT-III	105.00 ± 18.79	104.46 ± 11.61	0.264	0.792
D-D	647.30 ± 1404.26	120.07 ± 71.55	4.108	*p* ≤ 0.001

D-D: D-dimer; TT: Thrombin Time; PT: Prothrombin Time; INR: International Normalized Ratio; PT%: Prothrombin Time Activity; APTT: Activated Partial Thromboplastin Time; FIB-C: Fibrinogen-c.

**Table 5:** Correlation analysis of coagulation indexes in patients with non-small-cell lung cancer.

Test items		PT	INR	PT%	APTT	TT	FIB-C	AT-III	D-D
PT	*r* value	1	0.998	-0.840	0.425	0.351	0.243	-0.399	0.450
	*p* value		*p* ≤ 0.001	*p* ≤ 0.001	0.019	0.057	0.196	0.002	0.013
INR	*r* value	0.998	1	-0.837	0.425	0.351	0.243	-0.404	0.445
	*p* value	*p* ≤ 0.001		*p* ≤ 0.001	0.019	0.057	0.195	0.002	0.014
PT%	*r* value	-0.840	-0.837	1	-0.355	-0.332	-0.209	0.329	-0.317
	*p* value	*p* ≤ 0.001	*p* ≤ 0.001		0.054	0.073	0.267	0.012	0.088
APTT	*r* value	0.425	0.425	-0.355	1	0.091	0.265	-0.128	0.116
	*p* value	0.019	0.019	0.054		0.632	0.042	0.501	0.543
TT	*r* value	0.351	0.351	-0.332	0.091	1	-0.439	-0.229	-0.120
	*p* value	0.057	0.057	0.073	0.632		0.001	0.022	0.529
FIB-C	*r* value	0.243	0.243	-0.209	0.265	-0.439	1	-0.056	0.148
	*p* value	0.196	0.195	0.267	0.042	0.001		0.768	0.436
AT-III	*r* value	-0.399	-0.404	0.329	-0.128	-0.229	-0.056	1	0.191
	*p* value	0.002	0.002	0.012	0.501	0.022	0.768		0.132
D-D	*r* value	0.450	0.445	-0.317	0.116	-0.120	0.148	0.191	1
	*p* value	0.013	0.014	0.088	0.543	0.529	0.436	0.132	

PT: Prothrombin Time; INR: International Normolized Ratio; PT%: Prothrombin Time Activity; APTT: Activated Partial Thromboplastin Time; TT: Thrombin Time; FIB-C: Fibrinogen-c; AT-?: Anti-Thrombin ?, D-D: D-dimer.

**Table 6:** Comparison of D-D levels in different age groups between the experimental group and the control group $\left(\bar{x}\pm \text{s}\right)$.

Test items	Experience group D-D (ng/ml)	Control group D-D (ng/ml)	*t* value	*p* value
30-59 age group	549.07 ± 771.86	75.04 ± 36.43	3.091	0.018
60-69 age group	1058.44 ± 1626.59	108.73 ± 43.41	4.437	0.014
70-79 age group	1194.02 ± 1566.64	145.15 ± 51.70	7.497	0.001
≥80 age group	764.68 ± 879.61	245.30 ± 85.92	3.579	0.016
Total	911.35 ± 1297.27	137.95 ± 82.35	7.091	*p* ≤ 0.001
*F* value	1.955	2.472		
*p* value	0.124	0.015		
*r* value	0.509	0.711		
*p* value	0.007	*p* ≤ 0.001		

D-D: D-dimer.

**Table 7:** Comparison of D-D levels between the experimental group and the control group $\left(\bar{x}\pm \text{s}\right)$.

Grouping	Experience group D-D (ng/ml)	Control group D-D (ng/ml)	*t* value	*p* value
Nonmetastasis	448.50 ± 505.17	120.07 ± 71.55	2.945	0.004
Metastasis	846.10 ± 1908.34		4.986	*p* ≤ 0.001
*t* value	4.560			
*p* value	0.001			

D-D: D-dimer.

**Table 8:** Comparison of the D-D level between the experimental group and the control group in different TNM classification $\left(\bar{x}\pm \text{s}\right)$.

Grouping TNM classification	Experience group		Control group		*t* value	*p* value
	Number	D-D (ng/ml)	Number	D-D (ng/ml)		
I stage	98	138.35 ± 36.54	950	120.07 ± 71.55	0.71	*p* > 0.05
II stage	101	200.55 ± 155.21			2.80	*p* < 0.05
III stage	172	671.36 ± 327.49			6.30	*p* < 0.05
IV stage	299	1500.85 ± 611.02			2.33	*p* < 0.05
*t* value	1.955					
*p* value	0.124					
*r* value	0.773					
*p* value	*p* ≤ 0.001					

D-D: D-dimer.

**Table 9:** *p* values of D-D level comparison between stage I and stage II in the experimental group.

	Grouping	I stage	II stage	III stage	IV stage
Grouping	D-D (ng/ml)	138.35 ± 36.54	200.55 ± 155.21	671.36 ± 327.49	1500.85 ± 611.02
I stage	138.35 ± 36.54	1	*p* < 0.05	*p* < 0.05	*p* < 0.05
II stage	200.55 ± 155.21	*p* < 0.05	1	*p* < 0.05	*p* < 0.05
III stage	671.36 ± 327.49	*p* < 0.05	*p* < 0.05	1	*p* < 0.05
IV stage	1500.85 ± 611.02	*p* < 0.05	*p* < 0.05	*p* < 0.05	1

D-D: D-dimer.

**Table 10:** Comparison of D-D levels between the experimental group and the control group before and after treatment (chemotherapy, targeted therapy) $\left(\bar{x}\pm \text{s}\right)$.

Grouping	Experience group D-D (ng/ml)	Control group D-D (ng/ml)	*t* value	*p* value
Before treatment	1077.26 ± 1301.52	120.07 ± 71.55	10.091	*p* ≤ 0.001
After treatment	669.69 ± 1014.35		7.241	*p* ≤ 0.001
*t* value	4.256			
*p* value	0.013			

D-D: D-dimer.

**Table 11:** Comparison of D-D levels before and after chemotherapy and targeted therapy in the experimental group $\left(\bar{x}\pm \text{s}\right)$.

Grouping	Chemotherapy group D-D (ng/ml)	Targeted therapy group D-D (ng/ml)	*t* value	*p* value
Before treatment	918.45 ± 1068.53	1168.54 ± 1437.67	0.294	0.863
After treatment	490.35 ± 674.18	898.49 ± 1295.33	5.131	0.011
*t* value	6.640	0.847		
*p* value	0.003	0.398		

D-D: D-dimer.

**Table 12:** Comparison of D-D levels between the experimental group and the control group before and after chemotherapy $\left(\bar{x}\pm \text{s}\right)$.

Grouping	Chemotherapy group D-D (ng/ml)	Control group D-D (ng/ml)	*t* value	*p* value
Before chemotherapy	918.45 ± 1068.53	120.07 ± 71.55	5.450	*p* ≤ 0.001
After chemotherapy	490.35 ± 674.18		0.988	0.023
*t* value	1.010			
*p* value	0.020			

D-D: D-dimer.

**Table 13:** Comparison of D-D levels in different chemotherapy cycles and before chemotherapy in the experimental group $\left(\bar{x}\pm \text{s}\right)$.

Grouping	After chemotherapy D-D (ng/ml)	Before chemotherapy D-D (ng/ml)	*t* value	*p* value
First cycle	669.52 ± 990.73	618.45 ± 1068.53	0.255	0.732
Second cycle	497.70 ± 507.63		0.748	0.079
Third cycle	371.94 ± 284.75		1.256	0.015
Fourth cycle	247.69 ± 152.09		1.377	0.013
*t* value	3.763			
*p* value	0.042			
*r* value	0.785			
*p* value	*p* ≤ 0.001			

D-D: D-dimer.

**Table 14:** *p* value of D-D level comparison between the experimental group and the chemotherapy cycle group.

	Grouping	Before chemotherapy	First cycle	Second cycle	Third cycle	Fourth cycle
Grouping	D-D (ng/ml)	918.45 ± 1068.53	669.52 ± 990.73	497.70 ± 507.63	371.94 ± 284.75	247.69 ± 152.09
Before chemotherapy	918.45 ± 1068.53	1	0.732	0.079	0.015	0.013
First cycle	669.52 ± 990.73	0.732	1	0.134	0.045	0.026
Second cycle	497.70 ± 507.63	0.079	0.134	1	0.176	0.044
Third cycle	371.94 ± 284.75	0.015	0.045	0.176	1	0.128
Fourth cycle	247.69 ± 152.09	0.013	0.026	0.044	0.128	1

D-D: D-dimer.

**Table 15:** Comparison of D-D levels between the experimental group and the control group before and after targeted therapy $\left(\bar{x}\pm \text{s}\right)$.

Grouping	Targeted therapy group D-D (ng/ml)	Control group D-D (ng/ml)	*t* value	*p* value
Before targeted therapy	1168.54 ± 1437.67	120.07 ± 71.55	7.497	*p* ≤ 0.001
After targeted therapy	898.49 ± 1295.33		0.713	0.174
*t* value	2.886			
*p* value	0.021			

D-D: D-dimer.

**Table 16:** Comparison of D-D levels in different targeted treatment cycles and before targeted treatment in the experimental group $\left(\bar{x}\pm \text{s}\right)$.

Grouping	After targeted therapy D-D (ng/ml)	Before targeted therapy D-D (ng/ml)	*t* value	*p* value
First cycle	1018.41 ± 1353.63	1168.54 ± 1437.67	0.433	0.206
Second cycle	969.42 ± 1482.26		0.735	0.093
Third cycle	854.86 ± 1321.79		0.959	0.046
Fourth cycle	709.83 ± 825.03		1.060	0.032
*t* value	8.939			
*p* value	*p* ≤ 0.001			
*r* value	0.887			
*p* value	*p* ≤ 0.001			

D-D: D-dimer.

**Table 17:** *p* value of D-D level comparison between the experimental group and the targeted treatment group.

	Grouping	First cycle	Second cycle	Third cycle	Fourth cycle
Grouping	D-D (ng/ml)	1018.41 ± 1353.63	969.42 ± 1482.26	854.86 ± 1321.79	709.83 ± 825.03
First cycle	1018.41 ± 1353.63	1	0.657	0.369	0.129
Second cycle	969.42 ± 1482.26	0.657	1	0.544	0.185
Third cycle	854.86 ± 1321.79	0.369	0.544	1	0.319
Fourth cycle	709.83 ± 825.03	0.129	0.185	0.319	1

D-D: D-dimer.

## Data Availability

Based on patient privacy, data are available from the corresponding authors on reasonable request.
